# An Efficient Ensemble Binarized Deep Neural Network on Chip with Perception-Control Integrated †

**DOI:** 10.3390/s21103407

**Published:** 2021-05-13

**Authors:** Wei He, Dehang Yang, Haoqi Peng, Songhong Liang, Yingcheng Lin

**Affiliations:** Chongqing Key Laboratory of Space Information Network and Intelligent Information Fusion, School of Microelectronics and Communication Engineering, Chongqing University, Chongqing 400030, China; hewei007@cqu.edu.cn (W.H.); dhyoung@cqu.edu.cn (D.Y.); penghaoqi@cqu.edu.cn (H.P.); shliang@cqu.edu.cn (S.L.)

**Keywords:** autonomous navigation engine, binarized deep neural network, energy efficiency, parallel computing, FPGA

## Abstract

Lightweight UAVs equipped with deep learning models have become a trend, which can be deployed for automatic navigation in a wide range of civilian and military missions. However, real-time applications usually need to process a large amount of image data, which leads to a very large computational complexity and storage consumption, and restricts its deployment on resource-constrained embedded edge devices. To reduce the computing requirements and storage occupancy of the neural network model, we proposed the ensemble binarized DroNet (EBDN) model, which implemented the reconstructed DroNet with the binarized and ensemble learning method, so that the model size of DroNet was effectively compressed, and ensemble learning method was used to overcome the defect of the poor performance of the low-precision network. Compared to the original DroNet, EBDN saves more than 7 times of memory footprint with similar model accuracy. Meanwhile, we also proposed a novel and high-efficiency hardware architecture to realize the EBDN on the chip (EBDNoC) system, which perfectly realizes the mapping of an algorithm model to hardware architecture. Compared to other solutions, the proposed architecture achieves about 10.21 GOP/s/kLUTs resource efficiency and 208.1 GOP/s/W energy efficiency, while also providing a good trade-off between model performance and resource utilization.

## 1. Introduction

Over the past few years, deep convolutional neural networks (DCNNs) have been extensively studied and applied due to their excellent performance [1], especially in the challenging field of computer vision, such as image classification [2,3], object detection [4,5], and instance segmentation [6,7]. Unfortunately, high computational cost and power consumption severely restrict the deployment of large-scale DCNNs on embedded mobile devices with limited resources and energy [8,9]. Therefore, more and more efforts focus on how to reduce the memory footprint and computational complexity of the DCNN models as much as possible while maintaining acceptable accuracy [10,11,12]. The research of Reference [13] shows a tempting and extreme binarized quantization scheme, which quantizes both weights and activations into 1-bit, and theoretically can reduce the model size by 32 times and significantly decrease the execution time compared to their floating-point counterparts.

Recently, many impressive works have been dedicated to the research of small autonomous unmanned aerial vehicles (UAVs) system which was equipped with DCNN models, aiming at making the UAVs avoid obstacles and drive autonomously in complex road environments (e.g., urban block), and remarkable results have been successfully achieved [14,15,16]. For instance, DroNet [14] is a residual convolutional neural network (CNN) which can predict the collision probability and control the flight angle of UAV. Autonomous UAVs will make a great contribution to social logistics, which brings great speed and convenience [17,18,19,20]. However, it is still a huge challenge to deploy large-scale DCNNs on chip in real-time applications with high computational performance requirements, due to the closed-loop control system based on DCNNs has high memory footprint and computational complexity [8]. Therefore, the DCNN based on binary spiking data coding has become a very promising solution, which can meet the design requirements of system on chip (SoC) in terms of lightweight, high energy efficiency, and inference performance. However, the binarized DCNNs with 1-bit weights and activations suffer from an unavoidable drop in the performance of accuracy owing to their weak ability of feature extraction and representation. Moreover, there will have been a further decrease in model accuracy because DCNNs that integrate control and vision computing have more complex outputs.

While a series of methodologies to improve the model performance of binarized DCNNs have recently been proposed in References [21,22,23]. Ensemble method [24] is a good way to tackle the challenges mentioned above, and is inspired by Reference [22], which discusses and compares the improvement of accuracy between a model in which data representation has more bits and another one with more single-bit networks. Our EBDN model considers aggregating several parallel reconstructed binarized DroNets to improve the performance accuracy of UAV system comparing to a single binarized model and it can simultaneously sense obstacles and control steering angle, which is very similar to the biological control mechanism of the brain [25]. Meanwhile, this paper presents a hardware architecture design of the ensemble binarized DroNet (EBDN) model for real-time UAV autonomous navigation engine, which integrates the capacity of perception and control. In addition, it is the novel approach developed for the deep neural network acceleration via model compression for efficient FPGA implementation.

In this paper, a novel and efficient architecture is proposed to implement EBDN on chip (EBDNoC) which is an extended version of our conference paper [26] that only verified on MNIST small dataset. In summary, this paper makes the following new contributions:

- We propose an EBDN model, which overcomes the accuracy bottleneck of a single network by integrating several binarized DroNets with ultra-low memory footprint, and can dramatically speed up the inference process of the model when compared to full precision neural networks. Moreover, the proposed method can be easily extended to other CNN designs.
- For our proposed EBDNoC system, we design a dedicated on-chip hardware streaming architecture with a fully pipelined data path and configurable degree of parallelism for ensemble architectures.
- We also evaluate the performance of our proposed hardware architecture on FPGA achieving comparable system throughput, energy and resource efficiency, while can provide the trade-off between model performance and hardware resource.

More detailed information will be explained in the rest of this paper, which is organized as follows. Section 2 introduces the related work about QNNs and FPGAs. Section 3 describes the proposed EBDN model. We demonstrate the hardware architecture design of EBDNoC in Section 4. Section 5 shows the comparable experiments and evaluations. Conclusions will be given in Section 6.

## 2. Related Work

### 2.1. Quantized Neural Networks

The scale of DCNN models and the amount of parameters required are expanding, which bring challenges to the deployment and application of deep neural network in the embedded edge devices. In response to these situations, the quantized neural network (QNN) methods can be useful for deep neural network acceleration and model compression.

In order to compress DCNN models and represent features by low precision, Wu et al. [27] proposed a quantization neural network architecture (Q-CNN) based on K-means clustering algorithm, which achieved better quantization results by reducing the estimation error of output response of each layer, and proposed an effective training scheme to suppress the multi-layer cumulative error after quantization. Vanhoucke et al. [28] proposed 8-bit fixed-point technology, by using the linear fixed-point method, the training speed of the model is increased by 2.5 times on the premise of little impact on the accuracy of the model. Gupta et al. [29] used stochastic rounding for network parameters, through 16-bit fixed-point representation, which can reduce the memory and floating-point operation and keep the classification accuracy. Dettmers [30] proposed an 8-bit approximation algorithm, which compresses the gradient value and activation value represented by the 32-bit floating point value to 8-bit, and reduced the error by dynamically determining the range of the exponent and decimal places. The Dorefa-net proposed by Zhou et al. [31] used low-bit gradients and activation values to train low-bit network connection weights, and realized the quantization of connection weights, activation functions, and inverse gradients.

The above quantization works are based on multi-bits, and there is also another series of methods on binary quantization neural networks which use only 1-bit in the inference stage, such as BinaryConnect [23] and XNOR-Net [21]. The quantization weights of BinaryConnect in the forward and backward of DCNNs used 1-bit fixed point instead of 32-bit floating-point. XNOR-Net provides an effective implementation of convolution operation by reconstructing a full precision filter with a single scale factor. The inference process of DCNNs they proposed allows hardware calculation to simplify multiplication operations and accumulation operations into XNOR bitwise operations and SHIFT operations. Although great progress has been made in binary neural network (BNN), it still faces huge performance loss compared with the full precision network, especially for lightweight networks.

### 2.2. DCNNs Deployed on FPGAs

Considering the DroNet inference stage, the traditional method [14] generally uses wireless network for image transmission and remote control after the base station completes the calculation, which greatly reduces the reliability and imposes restrictions on the maximum control distance. Therefore, Palossi et al. [16] proposed the deployment method of DroNet on the GAP8 chip to implement the navigation engine on MCU, but its parallelism is still not high enough and the computing efficiency is insufficient.

Recently, compared with CPU and GPU platform devices, FPGAs have become the accelerators [32] at the edge side for deep learning tasks because of their high performance, low power consumption, and reconfigurability. Song et al. [33] proposed a general purpose accelerator that tames the diversity of CNNs through using kernel-partition method. In terms of computing resources and transmission bandwidth aspects, Zhang et al. [34] proposed a design space exploration technique to increase the system throughput. Lu et al. [35] implemented a fast Winograd algorithm on FPGA, which can reduce the use of floating-point resources and greatly decrease the complexity of convolution operations. The study of Reference [36] proposed a specific hardware architecture to accelerate CNN dataflow, which maximizes resource utilization while minimizing the data communication.

In order to store the parameters directly on the chip memory without bandwidth restrictions, many studies aimed at accelerating binary neural networks are also proposed [37,38,39,40], they directly quantized both the weights and the activations into {−1, 1}, and replace multiplier accumulator (MAC) computation with XNOR-Popcount operation. FBNA architecture as the first fully binarized convolutional neural network accelerator was proposed in Reference [41], in which all layers are binarized, even including the input layer and padding.

In this work, we proposed the ensemble binarized DroNet (EBDN) model, which aggregates several single-bit networks to ensure accuracy performance and saves storage resources. The proposed circuit architecture of the EBDN model realized a high computational performance and ultra-low power consumption, which get benefit from its fully pipelined data structure and general-purpose parallelism design.

## 3. The Proposed Model

This section introduces the proposed network model, which is based on reconstructed DroNet model and combines both the methods of binarized and ensemble learning. We will describe the critical approaches separately.

### 3.1. Perception-Control Integrated Model

DroNet is introduced into our proposed EBDN model, which was initially presented by Loquercio et al. [14] and whose essential network topology is a lightweight residual CNN with two forked output layers. To realize autonomous visual navigation of UAV in the complex urban environment, DroNet skillfully controls the forward velocity of the UAV by collision probability of one output layer and utilizes steering coefficient of another layer after low-pass filtering to command the UAV’ yaw angle in flight. Finally, a non-uniform UAV system consists of a laptop that runs DroNet model and a commercial Parrot drone. The bidirectional communication between the laptop and the drone was realized through WIFI, which achieves wireless image transmission rate of 20 frames per second (FPS) from drone to laptop and transmits the control commands after processing the image from laptop to drone.

**Figure 1:**
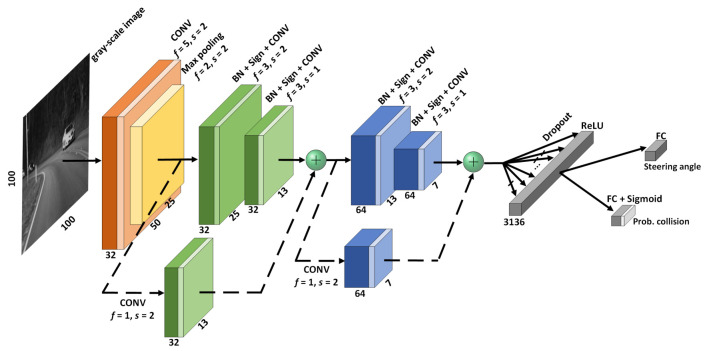
Network topology of EBDN model, in which essential architecture is a 6-layer network with 2 residual blocks and two separate fully-connected layers for judgement of steering angle and prediction of collision probability, respectively. The *f* as indicated above is the pooling or convolution kernel’s size, and the *s* is the corresponding stride.

**Figure 2:**
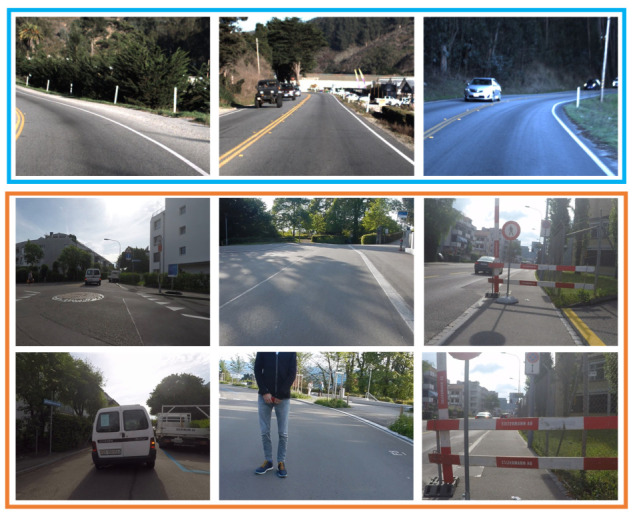
The sky blue box contains Udacity images which uses to learn and control steering angles, the orange box includes no-collision and corresponding collision frames which are collected to predict the probability of collision.

Our EBDN model is a perception-control integrated neural network based on DroNet, with two residual blocks [42] and two separated fully-connected (FC) layers, as is shown in Figure 1. Classification task for collision-avoidance and regression task for prediction of desired steering angle share all the parameters of the entire network except for the final outputs. Before the convolution (CONV) layer of each residual block, the input data will be normalized based on the distribution characteristics of batch feature data by the batch normalization (BN) operation [43], and then followed by a ReLU nonlinear function to enhance the fitting ability of network [44]. Residual networks become easier to optimize by adding shortcut connections, which have only a single residual convolutional (RCONV) layer. To achieve visual navigation and avoid the collision risk in UAVs systems, as is shown in Figure 2, two publicly available datasets Udacity (https://www.udacity.com/self-driving-car, accessed on 25 March 2020) for autopilot and Collision (http://rpg.ifi.uzh.ch/dronet.html, accessed on 25 March 2020) for collision prediction were used for training our model.

The two outputs of EBDN model, collision probability $p_{t}$ and predicted coefficient of steering $c_{t}$, are, respectively, applied to adjust the forward velocity $v_{t}$ and steering angle $\theta_{t}$ of UAV at the moment. The velocity constrained within [0, $v_{\max}$] will gradually increase to the maximum velocity with the decrease of collision probability. Furthermore, in order to filter out noise and obtain a smooth and continuous velocity, the low-pass filter is adopted into the approach which can be written as:

(1) $$v_{t}=\alpha \left(1-p_{t}\right)v_{\max}+\left(1-\alpha \right)v_{t-1}.$$

In fact, the predicted steering coefficient $c_{t}$ with a range [−1, 1] needs to be mapped to the actual steering angle in a range [$-\frac{\pi }{2}$, $\frac{\pi }{2}$] before it can be used to command the UAV, which can be expressed as follows:

(2) $$\theta_{t}=\beta \frac{\pi }{2}c_{t}+\left(1-\beta \right)\theta_{t-1},$$

where the default α and β are set to 0.7 and 0.5, respectively, and the maximum speed $v_{\max}$ can be set according to the experimental road environment.

Mean square error (MSE) and binary cross entropy (BCE) loss functions were used for training steering and collision prediction, respectively. However, simple superposition between two kinds of loss during the training process will greatly affect the optimization effect of training. Since the initial gradient of the regression task is much larger than that of the classification task, the learning approach of EBDN model will pay more attention to the loss of the classification task in the later stage of training, which can be represented by the following:

(3) $$L_{tot}=L_{MSE}+\max\left(0,1-\exp^{-decay(epoch-epoch_{0})}\right)L_{BCE},$$

where the default $epoch_{0}$ and decay factor decay are, respectively, set to 10 and $\frac{1}{10}$ in initial stage, and the weight of MSE loss is always 1. Moreover, more adjusted training details about EBDN are in Section 5.

### 3.2. Binarized Neural Network

Complex feature information can be extracted for difficult deep neural network tasks owing to the large-scale DCNN. Nevertheless, the large-scale DCNN model has more difficulty deploying on power-hungry and resource-limited embedded platforms due to the exponentially increasing resource requirements. Aiming at achieving high-performance and resource-efficient computing of perception-control integrated DCNN on the chip or edge device, the binarized way for data representation is introduced to drive the EBDN network.

After binarized network, we can fully store all weights on chip memory, and then convert −1 and 1 to 0 and 1 by affine transformation, respectively, which can convert original MAC operations to hardware-friendly XNOR and Popcount operations, i.e., performing bitwise XNOR between weights and inputs, and counting the number of 1 s in the intermediate result after XNOR operation. The convolution of above-mentioned binarized network with *n* input channels and *m* output channels can be defined as:

(4) $$Y_{m}=\sum_{i=1}^{n}sign\left(x_{i}\right)⊙sign\left(w_{i}\right)+b_{m},$$

where ⊙ indicates XNOR operation, $x_{i}$ and $w_{i}$, respectively, are i-th feature map input and associated weight, and $b_{m}$ is the neuron bias. The operation of batch normalization (BN) and binarized activation function follows the CONV layer, and $f_{act}\left(Y\right)$ is the sign function which is defined as:

(5) $$Y_{act}=f_{act}\left(Y\right)=sign\left(Y\right)=\left\{\begin{array}{cc} +1 & Y\geq 0\\ -1 & Y<0 \end{array}.\right.$$

This means that we can realize the nonlinear activation in the hardware architecture only by taking out the sign bits of intermediate results. The original definition of batch normalization operation is as follows:

(6) $$Y_{bn}=\frac{\gamma^{\left(i\right)}\left(Y_{m}-\mu^{\left(i\right)}\right)}{\sqrt{{\left[\sigma^{\left(i\right)}\right]}^{2}+\varepsilon }}+\beta^{\left(i\right)},$$

where σ, μ denote standard deviation and moving mean value of each i-th mini-batch (i.e., CONV layer’s outputs), and the trainable parameters γ, β represent scaling and shifting factor, which are used to adjust the variance of numerical distribution and the position of numerical mean, respectively. To facilitate hardware implementation, we can combine BN operation and activation, which can be expressed as:

(7) $$Z=sign\left(Y_{bn}\right)=sign\left(\gamma \left(\frac{Y_{m}-\mu }{\sigma }\right)+\beta \right),$$

where the $Y_{bn}$ represents the output result of BN layer, according to Reference [39], and Equation (7) can be equivalently transformed into the following expression:

(8) $$Z=sign\left(\frac{\gamma }{\sigma }\left(Y_{m}+\;\left(-\mu +\frac{\sigma }{\gamma }\beta \right)\right)\right).$$

In the inference phase, the γ and the σ constants parameters always keep positive, so that we just need to calculate the integer bias (−μ+(σ/γ)β), which called BN bias. In this way, we only need to use an adder to implement the BN and activation operations in the hardware circuit design.

### 3.3. Ensemble Learning Method

Due to the ultra-low computational complexity and memory requirements, binarized DCNNs have opened a new window for more embedded mobile devices to deploy the large-scale deep learning models. However, the accuracy of the binarized DCNN with 1-bit weights and activations will be severely decreased owing to its weak representation ability. In order to improve the performance of model accuracy, a binary ensemble neural network (BENN) has been proposed in Reference [22], which aggregates multiple single-bit binarized DCNNs using ensemble learning methods (e.g., bagging and boosting) to achieve better performance than multi-bit quantization networks. BENN also significantly enhanced the inherent stability and robustness of the original weak binarized DCNN, which was mainly reflected in the large fluctuation of training accuracy and model overfitting.

**Figure 3:**
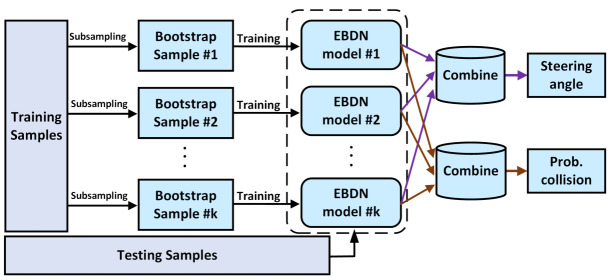
Schematic diagram of EBDN model. Each EBDN performs a separate training on each sample set generated by the bagging method and finally fuses all outputs through an ensemble mechanism to obtain the final results.

The bagging of ensemble method is introduced into our proposed model. The schematic diagram of bagging-based EBDN model is illustrated in Figure 3. In order to follow bagging’s bootstrapping principle [45], the *M* training samples of each EBDN model are randomly sampled and replaced from the total training dataset *D*, which are assumed to be an independent and identically distributed samples. After the independent training of *K*, we can get *K* weak EBDN models. In the inference stage, we use two different voting mechanisms, combined with the opinions of the k-EBDN model, to get the final prediction results. One is soft voting, which obtains the best output after averaging the probabilities of all predictive models, and the other is to select the labels with a majority of agreement as the final result, called hard voting.

**Figure 4:**
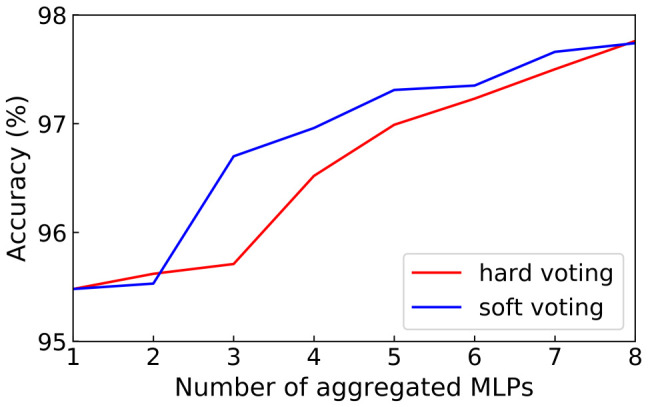
The accuracy of 64-128-128 BMLP models on MNIST when we change the number of aggregated BMLPs from 1 to 8.

We evaluate the performance of bagging method on 64-128-128 binarized multilayer perceptron (BMLP) for MNIST digital handwriting dataset, which is a three-layer fully connected neural network, and then we compare two voting mechanisms with different number of aggregated BMLPs in terms of model accuracy. As shown in Figure 4, The prediction accuracy of the model using soft voting was higher than that of the hard voting method. When BMLPs were aggregated from 1 to 8, the prediction accuracy increased from 95.5% to 97.7%. Nevertheless, our hardware architecture supports both hard voting and soft voting to meet the various task requirements of different networks, such as classification and regression.

We combine the bagging and binarized methods on the DroNet to form a unique EBDN model which is more suitable for deployment on edge devices, in which details of performance analysis and evaluation will be described in Section 5. Two hardware-friendly characteristics of the proposed EBDN model are as follows:

1. Compared to the original full-precision model, the EBDN model achieves a similar accuracy on Udacity and Collision datasets with much less memory footprint, which dramatically reduces the power consumption of the hardware. In addition, the binarized method eliminates the original inefficient CONV operation and replaces it with high energy-efficiency bitwise operation.
2. The flexible configurable number of subnetworks provides a trade-off between performance and resources for on-chip implementations, and, in fact, our network is suitable for many applications beyond the autonomous navigation of UAVs system mentioned in this paper.

## 4. Hardware Architecture Design

This section presents the hardware architecture of EBDNoC, an innovative data-stream-based fully pipelined circuit architecture for deploying ensemble networks. We will address the key details of the proposed architecture.

### 4.1. Overall Architecture

**Figure 5:**
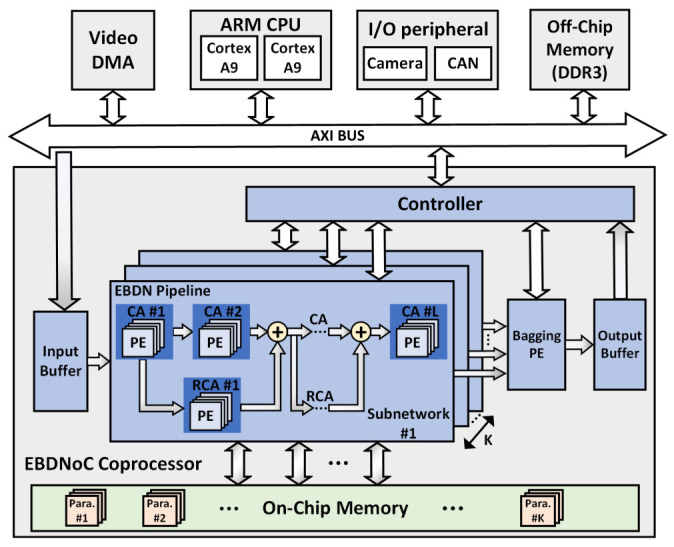
The overall architecture of EBDNoC, which mainly consists of two ARM cores, a neural network co-processor, and other peripherals. EBDNoC co-processor uses several Parallel pipelines to complete the ensemble calculation of the EBDN model. The bagging PE is in charge of aggregating the parallel outputs of EBDNPs.

The EBDNoC consists of several key components: two high-performance ARM Cortex-A9 processors, a EBDNoC neural network co-processor and other peripherals, including video direct memory access (VDMA) controller, off-chip memory system, and I/O peripherals. As shown in Figure 5, a Parallel pipeline-based architecture was used in EBDNoC co-processor, which primarily consist of *K* independent EBDN pipelines (EBDNPs) and a bagging processing element (PE). Each EBDNP performs the inference operation of one EBDN model which is defined as a subnetwork of the ensemble system, the bagging PE is responsible for aggregating the output results of *K* EBDNPs in the inference stage. Considering resource utilization and power consumption, we implemented the bagging method by integrating multiple parallel EBDNPs rather than reusing one EBDNP, which can load the parameters of different EBDN models from the block RAM (BRAM) memory at each inference phase, and it calculates the final outputs after each EBDN’s inferences are completed.

Each EBDNP has several computing arrays (CAs) cascaded through multiple PEs. Every CA is dedicated to the computation of corresponding macro-layer of EBDN model, commonly including CONV, BN, non-linear activation, and pooling operations. The residual computing arrays (RCAs) implement the inference of RCONV layer on the bypass of EBDN with the same circuit architecture as CA. ARM processor is used to flexibly configure the architecture of all CAs and control them through the Advanced eXtensible Interface (AXI) BUS. Benefiting from the low memory footprint of the EBDN model, all the parameters and intermediate results of the EBDN model are stored in the on-chip memory, which can eliminate the access bandwidth limitations of off-chip memory, greatly save power consumption, and improve system throughput.

Moreover, the I/O peripherals (e.g., controller area network (CAN) or universal asynchronous receiver/transmitter (UART)) mounted on the AXI BUS can build a EBDNoC prototype system, such as autonomous navigation UAV application. When the EBDNoC system is applied to image related tasks, it is controlled by ARM CPU and utilizes the video direct memory access (VDMA) IP core to complete high-speed and stable frame buffer, which is used to process image data transmission between the off-chip memory with very large capacity and the EBDNoC co-processor to improve the work efficiency of system.

### 4.2. Computing Array

**Figure 6:**
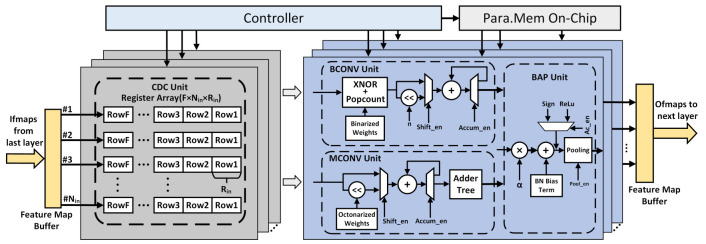
The block diagram of computing arrays, which consist of CDC unit, CONV unit, and BAP unit. The CDC unit is applied to cache input feature maps from last layer and generate convolution calculation data, CONV unit is responsible for convolution operation, the operations of batch normalization, activation, and pooling are performed in BAP unit.

The internal block diagram of CA is illustrated in Figure 6. All sub-modules adopt fully pipelined data path organization to reduce path delay and improve processing throughput. Meanwhile, we have implemented universality and configurability for the designs of our accelerator, which is controlled by the controller connected to the AXI BUS in each layer’s calculation. Different processing elements load the input feature maps (ifmaps) from the feature map buffer of last layer in a ping-pong way, and then compute the output feature maps (ofmaps) for each independent channel, and finally write the intermediate results to the feature map buffer for the next layer calculation. The most critical component of CA is *P* highly paralleled PE units, which are in charge of the computation of CONV and FC layer of EBDN model. The CA has several parts: convolution data cache (CDC) unit, CONV unit, and BAP (batch normalization, activation, and pooling) unit. We will reuse *P* PEs for multi-channel CONV computation, assuming that the CONV output channel of a macro-layer is $C_{out}$, and then $\frac{C_{out}}{P}$ computations are needed to get all the intermediate feature maps.

#### 4.2.1. Convolution Data Cache

The CDC unit is used to buffer the input data and generate the calculation data required by the subsequent CONV unit, which is composed of $N_{in}$ shift register buffer corresponding to the channels of ifmaps and each buffer contains sequential *F* rows of length $R_{in}$. To increase reconfigurability of the CDC unit, both $N_{in}$ and *F* are designed as configurable parameters, and the parameter $R_{in}$ can also be configured according to the ifmap size of each layer. *F* represents the convolution kernel’s size; therefore, a streaming structure is used to push the pixel data into the register buffer of every clock, and, after the *F* rows of ifmaps arrives, we can send the data to the CONV unit every clock. For another, if the architecture is not streaming, we can also load *F* rows of ifmaps from the feature map buffer per clock. In this case, we can further increase $R_{in}$ to improve the bandwidth of system at the expense of a few register resources. For example, we can set the $R_{in}$ to 56 when *F* is 3 and the size of ifmap is 28 × 28; in the first cycle, the ifmaps’ first six lines are required to calculate ofmaps’ first four lines so that it increases the bandwidth twice as much as when $R_{in}$ is 28. For FC layer, we only need to flatten register buffers into a one-dimensional vector and feed it into the CONV unit.

#### 4.2.2. CONV Unit

**Figure 7:**
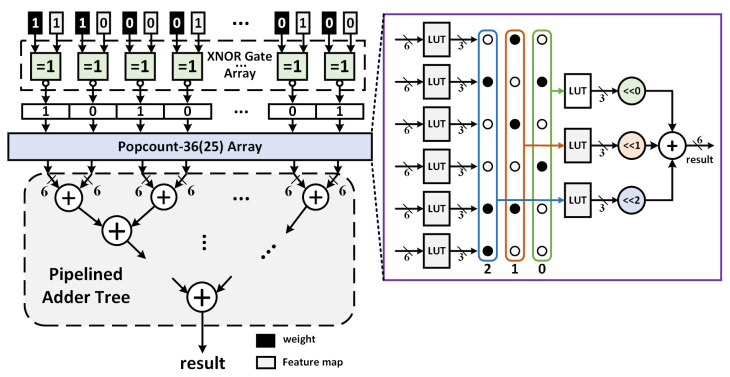
The overall architecture of XNOR-Popcount computing circuit unit, and the right purple block represents its internal architecture of Popcount-36(25) submodule.

To accommodate different quantization bits convolution calculation, the CONV unit is composed of BCONV unit and MCONV unit, which are responsible for the convolution operation of single bit and multi bit, respectively. The BCONV unit maps the original MAC operations of convolution to XNOR and Popcount operation. Figure 7 illustrates the structure of XNOR-Popcount module and the detailed logic implementation of Popcount-36 submodule. The cached data and weights are firstly computed by the XNOR gate array and sliced up several vectors of length 36, or maybe 25 for first layer because the size of convolution kernel is 5, and then followed by Popcount-36 array in which each Popcount-36 module calculates the Popcount value of an intermediate vector. The Popcount-36 operation depends on the 6-input 3-output look-up tables (LUTs) to make sufficient use of FPGA device resources at utmost. A Popcount-36 module consists of 6 LUTs, each of which performs a Popcount operation of a subvector of length 6. Subsequently, the value of each bit is calculated separately by the LUT similarly, and the weighted sum is obtained according to the bit weights by shifters and adder. It’s obvious that the vector length of the XNOR-Popcount module will be $L_{xp}=N_{in}\times F^{2}$ in the best case, which means that a PE can handle all the channels’ CONV windows of the previous layer at once. After the XNOR-Popcount operations, the intermediate results are send to the following shifter and accumulator. An accumulator is required for arbitrary network model because of PE cannot always complete the calculation at once for some networks which have the feature map with a large number of channels.

In addition, the PE with shifter and accumulator can meet the requirements of multi bit calculation, for example, the quantization precision of some layers is more than 1 bit, and the shifter can realize *N* shifts according to the binary bit weight to achieve the function of multiplier. The last operation is to send the previous results into the adder tree for accumulation.

#### 4.2.3. BAP Unit

The BN operation is accomplished by multiplier factor and integer addition, and, although we still utilize multipliers to achieve the multiplication between the factor α and previous result, we improve the reusability of PEs of each layer as much as possible, so that the multipliers remain at a low level. The binarized and ReLu activation can be selected by Ac_en signal according to the quantized bit. Then, the activation outputs flow into the Pooling unit, and it has a data cache module similar to the CDC unit, which is used to generate feature window data for pooling operations. It is noteworthy that the BAP unit can convert the max-pooling operation into a boolean OR operation when the activation value is only 0 or 1.

### 4.3. Memory System Design

#### 4.3.1. Quantization Strategy

A hybrid quantization strategy over all parameters we execute in EBDN model is given in Table 1 after we analyzed the training and feedforward process of its network. We choose a fixed representation Q = (BI,BF), using BI bits stand for the integer part, including sign bit and BF bits for the fractional part. 1-bit quantization method are implemented in the backbone network except for the first and last layer, and the RCONV layers, which can maintain the performance of the model with extra negligible resource and memory costs, because these layers have comparatively low computational and storage complexity. We only applied bias in the last two FC layers since we found that the bias term of CONV operation had almost no effect on model accuracy. Although there are many quantization layers over 1-bit, our hybrid quantization strategy still limits our model size to a dramatically small memory space, just 0.02 MB.

#### 4.3.2. Feature Map Buffer

**Figure 8:**
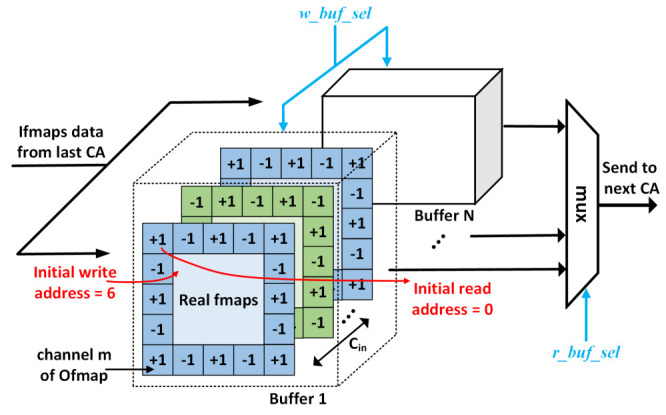
The structure of feature map buffer. The w_buf_sel and r_buf_sel signals are the choice signals for the write buffer and the read buffer, respectively.

Due to the PEs’ reuse of each CA, we must store the output feature maps of the previous layer for the CA module of the next layer to read repeatedly. As shown in Figure 8, the double-buffered mechanism is adopted in the storage architecture of feature map buffer (FMB), which can make the latter CA module read the feature maps like a pipeline operation to improve system processing speed. Inspired by Reference [41], we apply the odd-even padding operation by initializing the cache to corresponding value (±1) when the system is initialized because, compared with zero-padding, it can improve the performance of the model to a certain extent. Then, in the write phase, we only need to write the data one by one to the real feature maps area of cache specified by the controller, and the read address starts at 0 in the read phase. For example, when the size of the output feature maps requires 5 and the input feature size is 3, we need to have a padding operation with ±1 value around the input feature maps, and the initial read address and write address are 0 and 6, respectively.

#### 4.3.3. Memory Organization for Weights

All network parameters are stored in on-chip memory to eliminate the bandwidth limitations of external memory access, benefiting from the low memory footprint of the proposed EBDN model. Separate block RAM (BRAM) on the Xilinx ZYNQ device is used to store the CONV weights of each macro layer, while distributed RAM (DRAM) is used to store other parameters that take up less space, such as BN bias and multiplier factor. The width of each CONV weights memory block is equal to the sum of the *P* parallel PE widths of the current macro-layer (i.e., $L_{xp}\times P$), and the depth is equal to the number of cyclic computations $\frac{C_{out}}{P}$, which is mainly to extract multiple PE parameters with one clock to improve computing efficiency and processing speed. The organization of other parameters memory is similar.

### 4.4. Bagging PE

**Figure 9:**
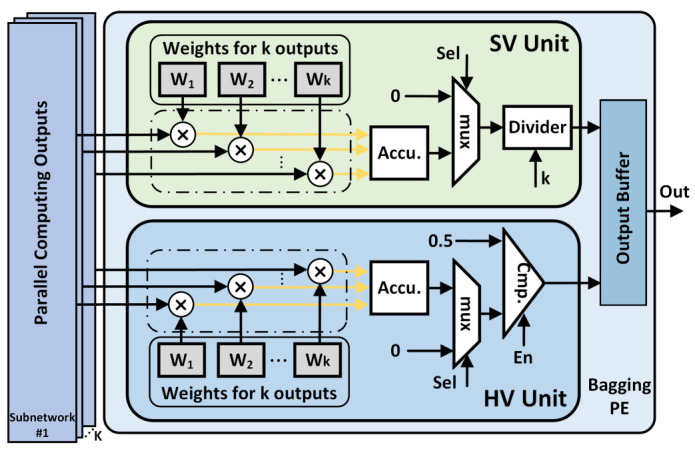
The hardware architecture design of bagging PE.

Figure 9 shows the hardware architecture of the bagging processing element (PE), which consists of soft voting (SV) unit and hard voting (HV) unit. The SV unit is responsible for computing the parallel outputs of steering angle from these CA modules, and it calculates the average value of steering angle after *K* accumulations. Meanwhile, we can allocate weights to each output according to the performance of each EBDN model, to obtain a better performance of the final result.

The HV unit performs the counting operation on the collision probability output of the EBDN model belonging to 0 or 1. In particular, to avoid all models with the same voting rights, the input of the accumulator has the weight we assign to each EBDN model based on its performance rather than 1. Finally, the weighted output is compared with the 0.5 threshold to get the final collision probability output.

## 5. Experiments and Results

In this section, the evaluation experiments can be divided into three parts. Firstly, we evaluate the performance of the EBDN model compared with other networks and then make a comparison between FPGA implementation and CPU, GPU platforms. Finally, we discuss and evaluate the efficiency of hardware architecture implemented on Xilinx Zynq 7Z100 FPGA device (Xilinx, San Jose, CA, USA), including throughput, resource utilization, and power consumption by testing and simulating on Vivado-2017 (Xilinx, San Jose, CA, USA).

### 5.1. Evaluation of EBDN Model

The bagging method was used to train and test the ensemble binarized DroNet on a publicly available dataset; it combines the Udacity’s dataset and the collected images which are associated with the collision probability according to the distance from the obstacles. The binarized neural network computing the gradient by floating-point value in the training process while binary value in the feedforward process, which leads to the slow convergence speed of loss function. Therefore, we improve the number of epochs for training from 100 to 200. Besides, to match the new training epochs, the initial decayed epoch $epoch_{0}$ and decay factor decay of BCE term’s weight in the mixed loss function that be expressed as Equation (3) were set as 20 and $\frac{1}{20}$, respectively.

Referring to Reference [46], we adopted Adam optimizer, in which the initial learning rate is $1^{-3}$ and the learning rate decay per epoch was set as $1^{-5}$. Based on these training settings and environments, multiple subnetworks are independently trained, and then were integrated the output of all subnetworks into one. We evaluated the steering regression task using explained variance ratio ($EVA=1-\frac{Var[y_{ture}-y_{pred}]}{Var[y_{ture}]}$) (EVA) and root-mean-squared error (RMSE) metrics, and the collision prediction task using average accuracy and F-1 score ($F-1=2\times \frac{precision\times recall}{precision+recall}$).

In addition to the binarized and ensemble methods, several details about network structure differ between the EBDN model and the original DroNet. The first difference is that we put the activation function after the CONV layer instead of before it, according to the rearrangement for binarized network’s macro-layer proposed in Reference [23]. Secondly, through comparative experiments, it is found that there are a large number of weights close to 0 in the third residual block convolution layers of DroNet. The sparsity of network will bring a small amount of network performance improvement, but with the doubling of the amount of computation, so the residual layer is removed in order to reduce hardware memory costs. The third is that we replace the original input image size of 200×200 with 100×100, which is convenient for unified implementation and hardly sacrifices the model accuracy.

**Figure 10:**
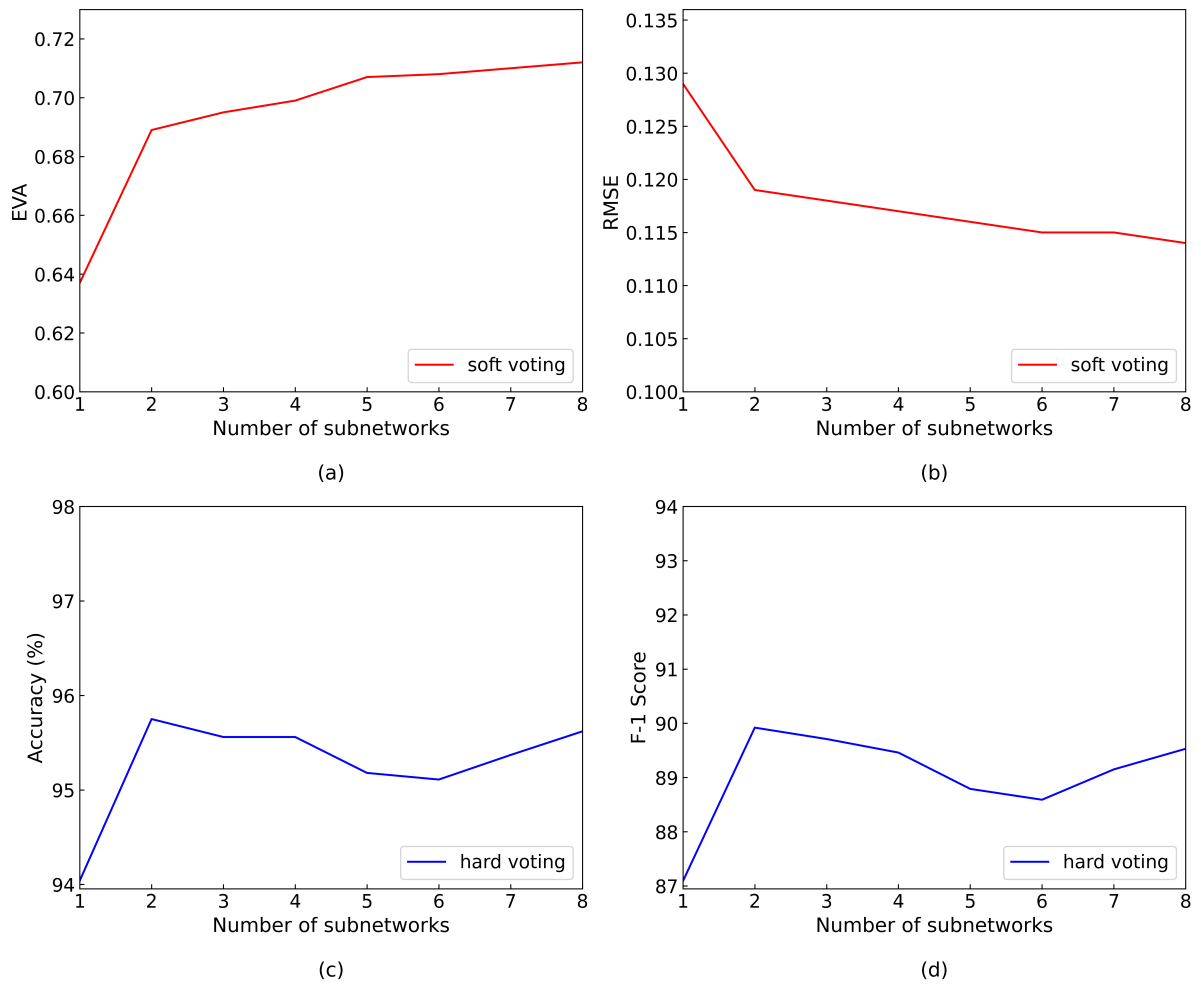
The performance of EBDN model variation as the number of aggregated subnetworks from 1 to 8. (**a**) EVA metric; (**b**) RMSE metric; (**c**) classification accuracy; (**d**) F-1 score.

Figure 10 shows the variation in the performance measurements of the EBDN model with the increasing number of aggregated weak subnetworks. Steering regression task is very difficult for low precision network model, but, with the help of ensemble learning technology, the performance of a single low precision network can still be improved very effectively. For the collision prediction task (i.e., classification task), we can clearly see that there is also a very significant improvement on both average accuracy and F-1 score metrics. In fact, as shown in Figure 10, the performance of regression task gains tend to level off when there are more than two subnetworks, which gives us a better trade-off between performance and resource consumption; thus, we can achieve a appreciable and practicable performance with only a few subnetworks.

As shown in Table 2, we compared our design with some classic models on model accuracy, memory footprint and inference speed. To ResNet-50 model [42], although the accuracy is better than all designs, our model achieves 41 times FPS and about 620 times lower cost of memory, its large memory footprint severely limits its deployment on embedded devices. Compared with VGG-16 [47], we achieve better performance about 24 times FPS and 179 times lower memory. For Giusti et al. [48], our design gets 12 times FPS and much better accuracy with less usage in memory footprint. Moreover, too low model performance can be very dangerous in real-time applications of UAVs. We only use 13% memory cost and achieve 14 times FPS and the similar accuracy with original DroNet [14]. All of the models mentioned above use the precision of 32-bit floating-point, while we have quantified our model in a hybrid approach of 1-bit and 8-bit for the hardware acceleration of EBDN model with low cost and high efficiency, so that our design can be easily applied to embedded devices. PULP-DroNet [15] is implemented with GAP8 SoC platform that has slightly higher performance. Compared with it, our design only uses quarter memory and get 16 times FPS, which shows the better potential in more complex scenarios with high demand for real-time computing capability. Overall, the proposed EBDN model hardly loses accuracy, while performance and memory cost are better than other models.

### 5.2. Comparison with CPU and GPU

In this subsection, we have achieved 285 frames/s on FPGA device with very low resource consumption to meet the needs of real-time applications and make a comparison between FPGA platform and three general platforms, including Intel Xeon Platinum 8269CY, Nvidia GTX2080 Ti, and Nvidia Jetson TX2. Both CPU and GPU evaluate the performance of DroNet with 32-bit floating-point precision on PC. The power consumption of the CPU comes from the Thermal Design Power (TDP) parameter on the data sheet, the GPU’s power is reported by *nvidia-smi* command, and then the power of TX2 and FPGA are obtained from on-board testing. We use FPS (frame/s) and FPS per watt (FPS/W) to represent the computing efficiency and energy efficiency, respectively. As shown in Table 3, our design gets a better performance of FPS about 9 times, and over 200 times lower power efficiency than CPU. For GPU platforms, although they achieved high FPS better than CPU, their computing energy efficiency is not efficient enough compared to FPGA.

### 5.3. Performance of Hardware Architecture

To evaluate the performance of our proposed hardware architecture design, the comparison between different scale CNN hardware implementations and the proposed design is shown in Table 4. We choose different CNN hardware designs from different CNN models, input image sizes and quantization strategies, and the selected small-scale networks in Reference [49] have only small input feature size (32 × 32), which can take advantage of low-precision design, such as 1-bit, to reduce storage resources consumption and improve system throughput. Therefore, most of the previous FPGA designs of binary neural network accelerators were limited to using very small data sets for verification, such as MNIST or Cifar-10. For large-scale deep network FPGA implementations [35,50,51], they choose to quantize the precision to 8 or 16 bits to ensure the accuracy of network. In this paper, the backbone of EBDN model is quantized using 1-bit weight and activation, and the input-output layer uses 8-bit precision. Then, we have analyzed the performance of EBDN model, which aggregated two subnetworks, because the system can achieve an excellent balance between model performance and hardware resource utilization, and the resource consumption is also a linear multiple of single subnetwork.

As presented in Table 4, the proposed design gets the performance of 439.1 average GOPS throughput with 208.1 GOPS/W energy efficiency. Compared to Reference [40], despite the large-scale network structure and binarized CNN architecture design, the throughput is still lower than our implementation; meanwhile, the BNN structure design of Reference [40] cannot support the other deep networks due to its weak scalability. Next, we further compare with the multi-bit quantization deep networks. In terms of References [35,50], its throughput outperforms the proposed design at the expense of the huge consumption of LUT resources. The research in Reference [35], whose hardware design is implemented by the Winograd algorithm, achieves 1.9 times throughput better performance than the proposed design result in the highest LUTs consumption and lower resource efficiency. The frequent off-chip memory access of the large-scale design in References [35,50,51] is the bottleneck of their design optimization, which are greatly limited by transmission bandwidth. Furthermore, the 8-bit and 16-bit precision designs consume much more DSPs than our proposed design in this paper.

Therefore, the proposed hardware architecture achieves comparable resource efficiency and energy efficiency performance owing to the novel ensemble binarized design and fully pipelined data path optimization. Meanwhile, the proposed design can provide a good trade-off between model performance and hardware resources by configuring parallelism or aggregating different number of subnetworks, which can be easily extended to other CNN structure.

## 6. Conclusions

This paper proposed the EBDN model, which is a perception-control integrated deep binarized DroNet model with a high energy efficiency and low hardware resource cost for visual navigation of UAV application. With the addition of binarized and ensemble learning methods, our algorithm methodology can not only bring the low storage and computational complexity but also overcome the challenge of poor performance of the low-precision model. The proposed EBDN model saves more than 13% of the memory footprint while achieving comparable performance with other state of the art models.

Aiming at the EBDN model, we proposed a high energy-efficiency and low-cost data-flow-driven hardware streaming architecture with high performance full-pipelined design on EBDNoC system, and implemented the prototype system of hardware design on Xilinx Zynq 7Z100 FPGA device with medium-scale resources. The proposed hardware design achieved area-efficiency and energy-efficiency of about 10.21 GOPS/kLUTs and 208.1 GOPS/W, respectively. Although our processor does not achieve the highest performance term, our hardware design can provide a trade-off between system throughput and resource consumption by setting different PE parallelism of each layer and configuring different number of subnetworks. Our future work will focus on multi-sensor-based and multi-channel visual navigation and diversified driving control model to fully utilize the system throughput of hardware architecture, and the reconfigurable design of network topology on hardware implementation. 

## Figures and Tables

**Table 1:** Quantization strategy for all parameters of each layer on EBDN model.

Layer	Weight	Activation	CONV Bias	BN Bias	Multiplier Factor	Mem.parameters (bits)
1 (CONV)	(0, 8)	(8, 0)	(0, 0)	-	-	6400
2 (CONV)	(1, 0)	(1, 0)	(0, 0)	(16, 0)	(16, 0)	10,240
3 (CONV)	(1, 0)	(1, 0)	(0, 0)	(16, 0)	(16, 0)	10,240
3 (RCONV)	(0, 8)	(8, 0)	(0, 0)	-	-	8192
4 (CONV)	(1, 0)	(1, 0)	(0, 0)	(16, 0)	(16, 0)	19,456
5 (CONV)	(1, 0)	(1, 0)	(0, 0)	(16, 0)	(16, 0)	38,912
5 (RCONV)	(0, 8)	(8, 0)	(0, 0)	-	-	16,384
6 (FC)	(0, 8)	(8, 0)	(0, 8)	-	-	25,096
6 (FC)	(0, 8)	(8, 0)	(0, 8)	-	-	25,096
Total	-	-	-	-	-	0.02 (MB)

**Table 2:** Comparison with other full precision models on performance of steering regression task and collision prediction task.

Model	[48]	[42]	[47]	[14]	[15]	EBDN (Ours)
EVA	0.672	0.795	0.712	0.737	0.748	0.712
RMSE	0.125	0.097	0.119	0.109	0.111	0.114
Avg. accuracy	91.2%	96.6%	92.7%	95.4%	95.9%	95.6%
F-1 score	0.823	0.921	0.847	0.901	0.902	0.900
Num. Layers	6	50	16	8	8	6
Memory (MB)	0.221	99.182	28.610	1.221	0.610	0.16
Precision	32-bit	32-bit	32-bit	32-bit	16-bit	1-bit, 8-bit
Speed (FPS)	23	7	12	20	18	285
Device	Intel Core i7	Intel Core i7	Intel Core i7	Intel Core i7	GAP8 SoC	Zynq 7Z100

**Table 3:** Comparison with CPU and GPU platforms.

Device	Intel Xeon Platinum 8269CY	Nvidia GTX2080 Ti	Nvidia Jetson TX2	Zynq 7Z100
Technology	14 nm	12 nm	16 nm	28 nm
Clock Freq. (MHz)	2.5 K	1.35 K	1.3 K	100
Precision	32 bits float	32 bits float	32 bits float	1 bit, 8 bits fixed
FPS (frame/s)	32.2	167.1	56.8	285.3
Speedup	1.0×	5.19×	1.76×	8.86×
Power (W)	205	53	4.56	6.48
Energy efficiency (FPS/W)	0.16	3.15	12.46	44.03

**Table 4:** Performance comparison of the proposed design with other previous CNN hardware architecture designs.

	Zhao [49]	Cho [40]	Zhang [50]	Lu [35]	Li [51]	Ours
FPGA Device	XC7Z020	XCZU7EV	XC7Z035	ZCU102	XC7Z100	XC7Z100
Frequency (MHz)	143	371	200	200	200	200
LUTs	46.9 K	4.8 K	82 K	600 K	136.9 K	43 K
DSPs	3	2	192	2520	1152	12
BRAMs	N/A	89	369	1824	912	286
Image Size	32 × 32	224 × 224	1024 × 1024	224 × 224	416×416	100 × 100
CNN Model	Cifar10	VGG-16	YOLOv2	Alexnet	VGG-16	EBDN
Precision	1-bit, 2-bit	1-bit	8-bit	16-bit	16-bit	1-bit, 8-bit
Throughput (GOPS)	207.8	177.68	111.5	854.6	452.8	439.1
Power (W)	4.7	0.711	5.96	23.6	19.52	2.11
Resource efficiency (GOPS/kLUTs)	4.43	37.02	1.36	1.424	3.31	10.21
Power efficiency (GOPS/W)	44.2	250	18.71	36.2	23.20	208.1

## Data Availability

Not applicable.

## Abbreviations

The following abbreviations are used in this manuscript:

EBDN	Ensemble Binarized DroNet
FPGA	Field Programmable Gate Array
DCNN	Deep Convolutional Neural Network
UAV	Unmanned Aerial Vehicle
SoC	System on Chip
BRAM	Block RAM
BNN	Binary Neural Network
VDMA	Video Direct Memory Access
